# Feeding practices for infants and young children during and after common illness. Evidence from South Asia

**DOI:** 10.1111/mcn.12222

**Published:** 2016-02-03

**Authors:** Kajali Paintal, Víctor M. Aguayo

**Affiliations:** ^1^ Regional Office for South Asia United Nations Children's Fund (UNICEF) Kathmandu Nepal

**Keywords:** child feeding, common childhood illnesses, diarrhoea, pneumonia, South Asia

## Abstract

Global evidence shows that children's growth deteriorates rapidly during/after illness if foods and feeding practices do not meet the additional nutrient requirements associated with illness/convalescence. To inform policies and programmes, we conducted a review of the literature published from 1990 to 2014 to document how children 0–23 months old are fed during/after common childhood illnesses. The review indicates that infant and young child feeding (IYCF) during common childhood illnesses is far from optimal. When sick, most children continue to be breastfed, but few are breastfed more frequently, as recommended. Restriction/withdrawal of complementary foods during illness is frequent because of children's anorexia (perceived/real), poor awareness of caregivers' about the feeding needs of sick children, traditional beliefs/behaviours and/or suboptimal counselling and support by health workers. As a result, many children are fed lower quantities of complementary foods and/or are fed less frequently when they are sick. Mothers/caregivers often turn to family/community elders and traditional/non‐qualified practitioners to seek advice on how to feed their sick children. Thus, traditional beliefs and behaviours guide the use of ‘special’ feeding practices, foods and diets for sick children. A significant proportion of mothers/caregivers turn to the primary health care system for support but receive little or no advice. Building the knowledge, skills and capacity of community health workers and primary health care practitioners to provide mothers/caregivers with accurate and timely information, counselling and support on IYCF during and after common childhood illnesses, combined with large‐scale communication programmes to address traditional beliefs and norms that may be harmful, is an urgent priority to reduce the high burden of child stunting in South Asia.

## Introduction

About a quarter (26%) of the world's children under five live in South Asia. Thirty‐eight per cent of them have stunted growth (UNICEF 2015). Stunting, or linear growth retardation during early childhood, is an outcome of biological and/or psychosocial deprivation (Stewart *et al*. 2013). The short‐term and long‐term consequences of stunting include impaired survival, physical growth and cognitive development in pre‐school age children; poor school readiness, school enrolment and learning outcomes in school‐age children; increased risk of obstetric complications and mortality in women; and reduced height, productivity and earnings in adults (Grantham‐McGregor *et al*. 2007; Walker *et al*. 2007; de Onis *et al*. 2013).

A significant proportion of stunting can happen prenatally. However, evidence indicates that most stunting in low‐income and middle‐income countries occurs during the first 24 months of life as a result of suboptimal breastfeeding and complementary feeding practices, often in combination with recurrent infections (Stewart *et al*. 2013; Jones *et al*. 2014). Furthermore, children's nutritional status can deteriorate rapidly during/after illness if the additional nutrient requirements associated with illness/convalescence are not met and nutrients are diverted from growth and development towards the immune response. Children's poor appetite induced by illness can contribute to perpetuate the vicious cycle of infection and stunting (Brown 2003; Ramachandran & Gopalan 2009; Gulati 2010; Neumann *et al*. 2012; Richard *et al*. 2014). Additionally, in low‐income and middle‐income countries, infant and young child feeding (IYCF) practices during and after common childhood illnesses can be particularly poor owing to harmful traditional practices and the low coverage/quality of primary health care services (Bhutta & Salam 2012; de Onis *et al*. 2012; Stewart *et al*. 2013).

Recognizing the importance of optimal IYCF practices for child survival, growth and development, the World Health Organization (WHO) launched in 2003 the *Global Strategy for Infant and Young Child Feeding* and issued in 2003 the *Guiding Principles for Complementary Feeding* of Breastfed and Non‐Breastfed Children (WHO/UNICEF 2003; WHO 2003a,2003b). These global frameworks highlight the importance of optimal IYCF practices during and after common childhood illnesses such as diarrhoea and pneumonia and emphasize the need to increase fluid intake during illness while feeding is maintained and increase food intake during convalescence. In addition, appropriate IYCF during and after illness is part of the WHO‐led *Global Strategy for the Integrated Management of Childhood Illnesses* (WHO 2005). The definition and measurement of the indicators for assessing IYCF practices – beyond the scope of this paper – are comprehensively detailed elsewhere (WHO 2008, 2010).

In South Asia, 1For the purpose of this paper, South Asia refers to the eight member countries of the South Asia Association for Regional Cooperation, namely Afghanistan, Bangladesh, Bhutan, India, Maldives, Nepal, Pakistan and Sri Lanka. breastfeeding is a quasi‐universal practice. An estimated 96% of children are breastfed at some point in their lives, and most (80%) continue to be breastfed at 2 years of age (Dibley *et al*. 2010; UNICEF 2015). However, data from household surveys across the region indicate that the majority of South Asian children are not fed as per the internationally agreed upon recommendations: only a quarter (27%) of newborns start breastfeeding within 1 h of birth; less than half (48%) of infants 0–5 months old are exclusively breastfed; only about half (56%) of infants 6–8 month olds are fed soft, semi‐solid or solid foods; and a mere 21% of children 6–23 months old are fed a diet that meets the minimum requirements in terms of feeding frequency and diet diversity (Senarath *et al*. 2012; UNICEF 2015). In view of this situation, researchers and practitioners have not hesitated to refer to IYCF in South Asia as a crisis (Memon 2012). There is evidence that the incidence and severity of common childhood diseases are high in this region (Walker *et al*. 2013). However, less is known about IYCF practices during and after common childhood illnesses in South Asia.

Thus, the objective of this paper is threefold: (1) document the current IYCF practices during and after common childhood illnesses – particularly diarrhoea, fever and pneumonia – and their trends since 1990 in South Asia; (2) document caregiver's behaviours and health providers' practices with respect to IYCF during and after common childhood illnesses in South Asia; and in light of the preceding objectives, (3) identify priorities in terms of policy formulation, programme design, research and advocacy to protect, promote and support optimal IYCF practices during and after common childhood illnesses in South Asia post 2015.

**Key messages**

- Information on infant and young child feeding (IYCF) behaviors and practices during common childhood illnesses in South Asia is limited. Information of IYCF after illnesses is virtually inexistent. The evidence available indicates that IYCF practices during common childhood illnesses are far from optimal.
- When sick, most children (up to 98%) continue to be breastfed although a significant proportion (up to 49%) are breastfed less frequently than usual. Few sick children (<20%) are breastfed more frequently than usual, as is recommended, to compensate for the additional fluid and nutrient requirements associated with illnesses.
- When sick, many children (up to 75%) see their complementary foods restricted in frequency, quantity and/or quality due to children's anorexia (perceived or real), lack of awareness of caregivers' about the feeding needs of sick children, traditional beliefs, or sub‐optimal counselling and support by health workers.
- In general, health providers do not advise mothers to increase breastfeeding frequency while encouraging sick children to eat soft, varied, and favourite foods during illness, as is recommended. Important policy, programme and capacity gaps exist with respect to IYCF for children during and after common childhood illnesses in many South Asian countries.

## Methods

We reviewed data and information from two primary sources: Demographic Health Surveys (DHS) and peer‐reviewed publications. DHS collects information on care‐seeking and care‐giving practices during diarrhoea, fever and pneumonia using standardized sampling methodologies, interview tools and data analyses procedures, with minor country‐specific adaptations. We reviewed the national DHS surveys conducted in South Asia between 1990 and 2014 to document the prevalence of common childhood illnesses – diarrhoea, fever and pneumonia – in children 0–23 months old, the frequency and type of medical advice that caregivers sought, the type of treatment and/or advice that children received and how children were fed during common childhood illnesses. For countries with two data points, trends in IYCF practices during and after common childhood illness were estimated as well as the average annual rate of improvement to quantify the average improvement in a given indicator per year between the base year and end year.

We also conducted a comprehensive review of the peer‐reviewed literature published between January 1990 and December 2014. Peer‐reviewed articles were identified through an online PubMed search using the following search terms and search filters: (1) search terms: <feeding>, <sick>, <morbidity>, <pneumonia>, <diarrhea/diarrhoea> and <IMCI/IMNCI>, each term combined with <Afghanistan>, <Bangladesh>, <Bhutan>, <India>, <Maldives>, <Nepal>, <Pakistan>, <Sri Lanka> and/or <Asia>; (2) search filters: age <child 0–59 months>; language: <English>; text availability: <abstract>; species :<human>; and search fields: <title/abstract>. Although children 0–23 months old are the focus of our analysis, we expanded our ‘child age’ search criteria to 0–59 months to capture additional publications that, while focusing on ‘children under five’ or ‘preschool‐age children’, also address IYCF practices in the first 2 years of life.

**Figure 1:**
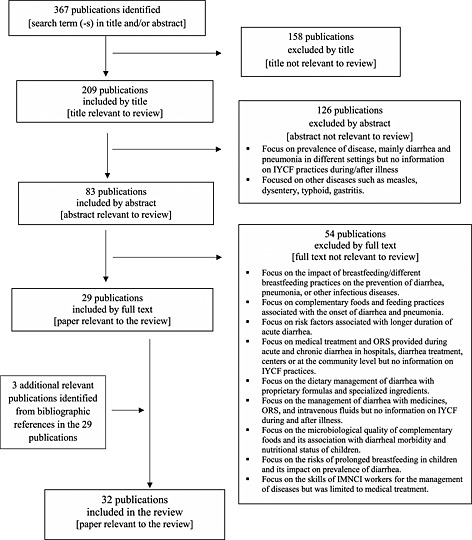
Flow diagram of literature review.

The PubMed search identified 367 publications with one or more of the search terms in the title and/or abstract. In‐depth scrutiny of the titles excluded 158 publications as not relevant to our review and identified 209 as *potentially relevant*. In‐depth scrutiny of the abstracts of these 209 publications excluded 126 as not relevant to our review and identified 83 as *likely relevant*. Lastly, full‐text scrutiny of these 83 publications excluded 54 as not relevant to our review and identified 29 articles that were *relevant* to our review. In addition, we reviewed the bibliographic references of these 29 papers to identify any additional publication that could have been missed by our online search and found three additional publications that were relevant to our analysis. Hence, 32 publications were included in our analysis as they focused specifically on IYCF practices during diarrhoea, fever and/or pneumonia in South Asian countries (Fig. 1).

In addition, we conducted interviews with 13 key informants. The purpose of the key informant interviews was not to collect key informants' views, opinions or recommendations but rather to help the authors of the paper identify the existing national policies, guidelines and programmes related to IYCF during and after common childhood illnesses in the eight countries included in the analysis. In the five large countries (Afghanistan, Bangladesh, India, Nepal and Pakistan), we interviewed two UNICEF staff by country, namely the Chief of Health and the Chief of Nutrition, while in the three smaller countries (Bhutan, Maldives and Sri Lanka), we interviewed one UNICEF staff per country, namely the Chief of the Health and Nutrition programme. This made a total of 13 key informants who in turn consulted with relevant national counterparts to complete the information‐gathering process.

## Findings

### Household survey evidence on infant and young child feeding and care practices during and after illness

Six countries – Bangladesh, India, Maldives, Nepal, Pakistan and Sri Lanka – had at least one DHS survey that included information on common childhood illnesses and IYCF practices (Afghanistan's 2010 DHS did not include data collection on child morbidity, and no DHS survey was available for Bhutan). DHS survey data indicate that in the countries included in the analysis, children 0–23 months old suffer from common childhood illnesses frequently. Up to 20–30% of the mothers/caregivers interviewed reported that their children had suffered from diarrhoea or pneumonia in the 2 weeks prior to the survey. The prevalence of common childhood illnesses – diarrhoea, fever and pneumonia – was highest in Pakistan. In all countries, the prevalence of fever was higher than the prevalence of diarrhoea or pneumonia. Similarly, in all countries, the prevalence of common childhood illnesses was lowest during the exclusive breastfeeding period (0–5 months) and highest during the early complementary feeding period (6–11 months) (Table 1).

**Table 1:** Number and percentage of children 0–23 months old who experienced diarrhoea, fever or pneumonia in the 2 weeks preceding the survey (South Asia, Demographic and Health Surveys)

	Prevalence of common childhood diseases			
	*n*	Diarrhoea (%)	Fever (%)	Pneumonia (%)
Bangladesh, 2011				
0–5 months	816	3.1	35.1	6.2
6–11 months	864	8.4	49.2	7.4
12–23 months	1 547	7.1	42.6	6.9
0–23 months	3 227	6.4	42.5	6.9
India, 2006				
0–5 months	5 127	10.6	11.6	6.2
6–11 months	5 276	18.1	21.1	8.1
12–23 months	10 419	13.8	19.1	7.1
0–23 months	20 822	14.1	17.8	7.1
Maldives, 2009				
0–5 months	406	2.5	21.8	—
6–11 months	441	6.9	34.4	—
12–23 months	822	6.7	33.7	—
0–23 months	1 669	5.7	31.0	<1
Nepal, 2011				
0–5 months	531	12.9	17.1	3.9
6–11 months	491	24.1	29.7	7.5
12–23 months	1 000	23.9	24.2	7.9
0–23 months	2 022	21.1	23.7	6.8
Pakistan, 2012				
0–5 months	1 164	25.8	33.8	15.3
6–11 months	1 024	35.3	49.8	21.2
12–23 months	2 074	32.9	46.4	20.2
0–23 months	4 262	31.5	43.8	19.1
Sri Lanka, 2007				
0–5 months	634	1.5	9.5	2.2
6–11 months	739	9.1	22.8	4.9
12–23 months	1 438	4.7	21.5	5.0
0–23 months	2 811	5.1	19.1	4.3

The proportion of caregivers who sought medical advice in the event of diarrhoea was highest in India and Pakistan (>60%); the proportion of caregivers who sought medical advice in the event of fever was highest in Maldives and Sri Lanka (>80%); lastly, the proportion of caregivers who sought medical advice in the event of pneumonia was highest in India and Pakistan (>65%). The lowest proportion of caregivers seeking medical advice for common childhood illnesses was recorded in Bangladesh (30.1%, 31.4% and 41.4% for diarrhoea, fever and pneumonia, respectively (Table 2).

**Table 2:** Among children 0–23 months old who experienced diarrhoea, fever or pneumonia in the 2 weeks preceding the survey, number and percentage for whom advice/treatment was sought from a health facility or health provider (South Asia, Demographic and Health Surveys)

	Bangladesh 2011	India 2006	Maldives 2009	Nepal 2011	Pakistan 2012	Sri Lanka 2007
	*N* (%)	*N* (%)	*N* (%)	*N* (%)	*N* (%)	*N* (%)
Diarrhoea						
0–5 months	25 (43.6)	542 (57.1)	—	68 (32.6)	300 (60.0)	—
6–11 months	73 (30.1)	956 (60.3)	—	118 (41.6)	361 (60.8)	—
12–23 months	109 (27.0)	1434 (66.1)	—	239 (40.2)	682 (68.0)	—
0–23 months	207 (30.1)	2932 (62.5)	—	425 (39.4)	1343 (64.3)	—
Fever						
0–5 months	155 (36.0)	593 (71.0)	88 (79.9)	91 (34.2)	394 (66.7)	60 (62.5)
6–11 months	284 (32.8)	1113 (76.4)	152 (86.2)	146 (45.9)	51 (64.9)	169 (87.5)
12–23 months	466 (29.0)	1991 (71.4)	277 (84.5)	242 (46.2)	962 (66.0)	310 (85.5)
0–23 months	905 (31.4)	3697 (72.8)	517 (84.2)	479 (43.8)	1407 (66.2)	539 (83.6)
Pneumonia						
0–5 months	51 (39.8)	319 (70.7)	—	—	178 (70.7)	14 (0.0)
6–11 months	64 (42.8)	427 (76.9)	—	—	217 (62.7)	37 (67.6)
12–23 months	106 (41.4)	743 (69.0)	—	—	420 (65.5)	71 (65.7)
0–23 months	221 (41.4)	1489 (71.6)	—	—	815 (65.9)	122 (58.7)

Data on the type of treatment/care provided to children 0–23 months old who experienced diarrhoea, fever or pneumonia in the 2 weeks preceding the survey and for whom advice or treatment was sought from a health facility or health provider were available for Bangladesh, India, Nepal and Pakistan. The proportion of children who received oral rehydration solutions (ORS) or increased fluids was highest in Bangladesh (>75%) and lowest in India (<20%) (Table 3). Similarly, the proportion of children who received antibiotic therapy for the treatment of fever and pneumonia was highest in Bangladesh (>66%) and lowest in India (<15%) (Table 4).

**Table 3:** Among children 0–23 months old who experienced diarrhoea in the 2 weeks preceding the survey and for whom advice or treatment was sought from a health facility or health provider, percentage according to the type of treatment/care that they were provided during the diarrhoea episode (South Asia, Demographic and Health Surveys)

	ORS	RHF	Children given ORS or RHF	Children given increased fluids	Zn treatment			
					Zn syrup	Zn tablet	Zn supplements	Zn + ORS
Bangladesh, 2011								
0‐5 months	46.1	0.0	46.1	1.7	13.9	7.2	—	8.8
6–11 months	73.4	9.3	76.2	23.0	39.4	21.9	—	35.8
12–23 months	75.7	7.1	77.7	25.5	32.0	23.2	—	39.7
0–23 months	71.3	9.8	73.4	21.7	32.4	21.5	—	34.6
India, 2006								
0–5 months	13.7	15.6	15.6	2.6	—	—	0.0	—
6–11 months	21.3	15.9	31.8	7.9	—	—	0.5	—
12–23 months	34.6	23.6	48.0	11.1	—	—	0.3	—
0–23 months	26.4	20.8	36.7	8.5	—	—	0.3	—
Nepal, 2011								
0–5 months	5.8	—	5.8	13.6	—	—	1.8	0.0
6–11 months	35.2	—	35.2	9.3	—	—	5.7	4.6
12–23 months	48.2	—	48.2	17.7	—	—	6.3	6.2
0–23 months	37.8	—	37.8	14.7	—	—	5.4	4.8
Pakistan, 2012								
0–5 months	25.9	3.3	27.2	7.9	—	—	0.6	—
6–11 months	38.8	10.5	42.7	5.3	—	—	0.3	—
12–23 months	44.4	11.0	48.5	8.2	—	—	2.7	—
0–23 months	38.8	23.6	42.2	7.4	—	—	1.6	—

ORS, oral rehydration solution; RHF, recommended home fluids.

**Table 4:** Among children 0–23 months old who experienced fever or pneumonia in the 2 weeks preceding the survey and for whom advice or treatment was sought from a health facility or health provider, percentage according to the type of treatment/care that they were provided during the fever/pneumonia episode (South Asia, Demographic and Health Surveys)

	No. of children with fever	Percentage of children with fever who received antimalarial drugs	Percentage of children with fever who received antibiotic drugs	No. of children with pneumonia	% of children with pneumonia who received antibiotic drugs
Bangladesh, 2011					
0–5 months;	286	1.8	54.3	51	69.1
6–11 months	425	0.1	66.9	64	81.8
12–23 months	659	0.9	70.7	106	78.0
0–23 months	1 370	0.8	66.1	221	77.0
India, 2006					
0–5 months	5 127	7.5	14.6	319	14.6
6–11 months	5 276	7.2	14.9	427	11.9
12–23 months	10 419	9.2	13.8	743	12.7
0–23 months	20 822	8.3	14.3	1489	12.9
Nepal, 2011					
0–5 months	91	1.8	29.4	—	—
6–11 months	146	0	34.4	—	—
12–23 months	242	1.5	38.1	—	—
0–23 months;	479	1.1	35.3	—	—
Pakistan, 2012					
0–5 months	394	2.4	32	178	41.4
6–11 months	51	5.3	41.1	217	44.1
12–23 months	962	4.0	40.0	420	43.5
0–23 months	1 407	3.6	37.8	815	43.2

In these four countries – Bangladesh, India, Nepal and Pakistan – DHS information on IYCF practices during/after common childhood illnesses focused only on feeding practices during diarrhoea (Table 5). No information was available on IYCF practices when children had fever or pneumonia or after episodes of diarrhoea, fever or pneumonia. The proportion of mothers/caregivers who fed their children more/same fluids as usual was highest in Bangladesh (72.6%) and lowest in India (Table 5).

**Table 5:** Percentage distribution of children 0–23 months old who had diarrhoea in the 2 weeks preceding the survey by amount of liquids and food offered during the diarrhoea episode compared with normal practice as reported by the mother/primary caregiver (South Asia, Demographic and Health Surveys)

	Bangladesh 2011		India 2006		Nepal 2011		Pakistan 2012	
	Liquids	Solids	Liquids	Solids	Liquids	Solids	Liquids	Solids
	(%)	(%)	(%)	(%)	(%)	(%)	(%)	(%)
Children 0–5 months								
More	1.7	0.0	2.6	1.7	13.6	0.0	7.9	0.9
Same as usual	71.9	50.1	58.7	23.3	69.0	10.0	59.4	12.4
Somewhat less	16.5	16.5	19.4	11.9	2.2	6.0	19.2	8.8
Much less	5.3	5.3	7.0	6.9	0.0	3.4	6.7	6.9
None	4.6	7.7	12.0	0.7	15.1	0.7	6.5	0.7
Never gave food	—	—	—	54.4	—	79.9	—	70.2
Do not know or missing	—	—	—	1.0	—	0.0	—	0.0
Children 6–11 months								
More	23.0	14.9	7.9	1.9	9.3	5.5	5.3	1.4
Same as usual	44.5	40.0	48.3	30.3	78.3	58.4	53.7	34.6
Somewhat less	25.1	29.4	28.4	25.2	5.3	8.2	32.0	23.5
Much less	7.0	9.3	9.7	7.4	0.0	1.9	6.5	6.4
None	0.4	1.1	5.4	5.6	7.1	0.7	1.5	7.7
Never gave food	—	20.4	—	29.2	—	25.3	—	25.5
Do not know or missing	—	—	0.4	0.3	0.0	0.0	0.8	0.8
Children 12–23 months								
More	25.5	12.5	11.1	1.7	17.7	10.1	8.2	2.7
Same as usual	50.3	54.7	45.7	39.7	67.5	65.5	52.8	44.5
Somewhat less	21.8	23.8	29.8	34.2	11.4	21.8	31.3	36.1
Much less	2.4	4.4	10.6	13.6	1.1	2.1	7.5	9.9
None	—	4.5	2.4	4.0	2.4	0.5	0.3	3.9
Never gave food	—	0.2	—	6.0	—	0.0	—	3.0
Do not know or missing	—	—	0.4	0.9	0.0	0.0	0.0	0.0
Children 0–23 months								
More	21.7	11.8	8.5	1.8	14.7	7.2	7.4	1.9
Same as usual	50.9	49.0	49.0	33.6	70.7	54.6	54.5	34.7
Somewhat less	22.3	24.9	27.4	27.1	8.2	15.5	28.8	26.6
Much less	4.4	6.2	9.6	10.3	0.6	2.3	7.1	8.3
None	0.7	3.4	5.2	5.1	5.7	1.0	2.0	5.6
Never gave food	—	1.1	—	0.4	—	0.4	—	0.3
Do not know or missing	0.0	0.0	0.0	0.1	0.0	0.0	0.0	0.0

We were able to examine time trends in four countries – Bangladesh, India, Nepal and Pakistan, where three DHS surveys were available for the period 1990–2014. Bangladesh and Nepal made significant progress in reducing the prevalence of diarrhoea, fever and pneumonia in children 0–23 months old, mirrored by significant increases in care‐seeking behaviour for these common childhood illnesses. Improvements in India were low to nil, while surveys in Pakistan reported a significant deterioration (Table 6).

**Table 6:** Percentage of children 0–23 months old who experienced diarrhoea, fever or pneumonia in the 2 weeks preceding the survey and for whom advice/treatment was sought from a heath facility or health provider (Demographic Health Surveys, 1990–2013)

Bangladesh	1994	2004	2011	AARI*
Children 0–23 months old with diarrhoea (%)	13.2	10.1	6.4	−0.40
Children with diarrhoea seeking medical advice (%)	20.6	18.8	30.1	+0.56
Children 0–23 months old with fever (%)	—	46.4	42.5	−0.56
Children with fever seeking medical advice (%)	—	22.8	31.4	+1.23
Children 0–23 months old with pneumonia (%)	26.8	26.9	6.9	−1.17
Children with pneumonia seeking medical advice (%)	29.3	25.4	41.4	+0.71
India	1992	1998	2006	AARI*
Children 0–23 months old with diarrhoea (%)	13.2	21.1	14.1	+0.06
Children with diarrhoea seeking medical advice (%)	61.9	61.5	62.5	+0.04
Children 0–23 months old with fever (%)	22.8	30.3	17.8	−0.36
Children with fever seeking medical advice (%)	67.7	—	72.8	+0.36
Children 0–23 months old with pneumonia (%)	7.4	20.2	7.1	−0.02
Children with pneumonia seeking medical advice (%)	68.3	63.6	71.6	+0.24
Nepal	1996	2006	2011	AARI*
Children 0–23 months old with diarrhoea (%)	31.2	18.2	21.1	−0.67
Children with diarrhoea seeking medical advice (%)	14.1	27.1	39.4	+1.69
Children 0–23 months old with fever (%)	41.2	21.5	23.7	−1.17
Children with fever seeking medical advice (%)	—	33.1	43.8	+2.14
Children 0–23 months old with pneumonia (%)	37.8	7.2	6.8	−2.07
Children with pneumonia seeking medical advice (%)	18.3	42.8	—	+2.45
Pakistan	1991	2007	2012	AARI*
Children 0–23 months old with diarrhoea (%)	19.2	31.6	31.5	+0.59
Children with diarrhoea seeking medical advice (%)	51.0	57.4	64.3	−0.01
Children 0–23 months old with fever (%)	35.6	36.4	43.8	+0.39
Children with fever seeking medical advice (%)	66.4	68.6	66.2	−0.01
Children 0–23 months old with pneumonia (%)	19.1	15.9	74.1	+0.00
Children with pneumonia seeking medical advice (%)	68.4	74.1	65.9	−0.12

* Average annual rate of improvement (AARI) quantifies the average rate of change between base year and end year.

Table 7 summarizes the trends in feeding and care practices for children 0–23 during diarrhoea episodes. Over the 1990–2014 period, the proportion of children with diarrhoea who were given Oral Rehydration Solution (ORS) increased in Bangladesh, India and Nepal, while there was no improvement in Pakistan. The proportion of children who were not given ORS/recommended home fluids/increased fluids declined in all countries. The highest average annual rate of reduction was recorded in Bangladesh (0.41) and the lowest in India (0.11). Detailed information on trends in IYCF during diarrhoea was available only for Nepal (2006–2011) and Pakistan (2007–2012). In both countries, most mothers reported that the amount of liquids offered to their infants during the diarrhoea episode was ‘same as usual’ in both base year and end year. Only about half the mothers in Nepal and one‐third of mothers in Pakistan reported that the amount of food offered to their children was ‘same than usual’ – with no improvement between base year and end year.

**Table 7:** Percentage of children 0–23 months old who experienced diarrhoea in the 2 weeks preceding the survey, by type of treatment/care they were provided and amount of liquids/food offered compared with normal practice (Demographic Health and Surveys, 1990–2013)

Bangladesh	1994	2004	2011	AARI
Children given ORS (%)	47.8	65.2	71.3	1.38
Children given recommended home fluids (%)	14.3	17.9	7.0	−0.43
Children given increased fluids (%)	48.8	48.6	21.7	−1.59
Children not given ORS/recommended home fluids /increased fluids (%)	31.5	16.9	24.5	−0.41
India	1992	1996	2006	
Children given ORS (%)	17.8	25.5	26.4	0.61
Children given recommended home fluids (%)	18.1	12.8	17.4	−0.05
Children given increased fluids (%)	13.8	20.7	8.5	−0.38
Children not given ORS/ recommended home fluids /increased fluids (%)	61.4	54.3	59.8	−0.11
Nepal	1996	2006	2011	
Children given ORS (%)	23.8	25.5	37.8	0.93
Children given recommended home fluids (%)	4.2	—	—	—
Children given increased fluids (%)	33.1	20.1	14.7	−1.23
Children not given ORS/recommended home fluids/increased fluids (%)	53.8	62.6	51.6	−0.15
Feeding practices: amount of liquids offered to children (%)				
More		20.1	14.7	−1.08
Same as usual		63.4	70.7	1.46
Less than usual		13.4	8.8	−0.92
None		3.1	5.7	0.52
Feeding practices: amount of food offered to children (%)				
More		4.7	7.2	0.50
Same as usual		53.3	54.6	0.26
Less than usual		25.1	17.8	−1.46
None		2.6	1.0	−0.32
Never gave food		0.2	0.4	0.04
Pakistan	1991	2007	2012	
Children given ORS (%)	38.8	40.3	38.8	0.0
Children given recommended home fluids (%)	14.7	15.8	9.1	−0.27
Children given increased fluids (%)	9.4	18.2	7.4	−0.10
Children not given ORS/recommended home fluids/increased fluids (%)	54.9	46.7	49.7	−0.25
Feeding practices: amount of liquids offered to children (%)				
More		18.2	7.4	−2.16
Same as usual		43.3	54.5	2.24
Less than usual		33.1	35.9	0.56
None		5.2	2.0	−0.64
Do not know or missing		0.2	0.0	0.0
Feeding practices: amount of food offered to children (%)				
More		6.3	1.9	−0.88
Same as usual		34.2	34.7	0.10
Less than usual		31.1	34.9	0.76
None		4.8	5.6	0.16
Never gave food		23.3	0.3	−4.6
Do not know or missing		2.0	0.0	0.0

AARI, average annual rate of improvement; ORS, oral rehydration solution.

**Table 8:** Summary table of findings from review of evidence on infant and young child feeding during and after common childhood illnesses in South Asia (1990–2014)

Findings	Disease	Breastfeeding (BF)	Fluid intake	Complementary foods (CF) & feeding practices	Traditional beliefs and their role in feeding practices	Community elders/traditional practitioners' advice	Health professionals’ advice	Interpersonal and group counselling
Study								
Agha *et al.,* 2007 Pakistan	Diarrhea ARI	NA	‐ 88% mothers continued fluids,	‐ Continued feeding: 17% for Diarrhea & 56% for pneumonia,	NA	NA	NA	NA
			‐ 11.7% mothers gave less fluid for diarrhea and lesser for pneumonia,	‐ Less food: 43.9% children (diarrhea) and 83% with pneumonia,				
			‐ Caregivers are less resistant to giving more fluids than to food (diarrhea). This is due to emphasis of fluids than food in media campaigns.	‐ Mothers gave “butter” to help cure diarrhea.				
Ahmed *et al.,* 1992 Bangladesh	Diarrhea	‐ EBF is increased for children < 1 years from 14.8% to 35.2%,	NA	‐ 22% had normal family diets,	‐ 50% mothers withheld some food believing ‐ “Some foods cause diarrhea, others increase frequency of loose motion, & withholding or adding some food cures illnesses.”	‐ Elders in the family advised mothers on care of sick child.	NA	NA
		‐ Mothers partially BF decreased 62.5% to 43.2%,		‐ 23.7% mothers believed that withholding or adding certain foods can cure diarrhea.				
		‐ Increase in exclusive BF due to withholding of other foods.						
					‐ These children were given special diets.			
Badruddin *et al.,* 1991 Pakistan	Diarrhea	‐ >95% BF continued.	NA	‐ Withholding CF: None,	‐ Buffalo milk commonly given,	NA	NA	NA
				‐ 25% decreased food intake due to poor appetite and decreased food intake,	‐ Home remedies, herbal medicines & teas were given to children with diarrhea.			
				‐ Nature & amount of fluids & foods varied according to disease intensity & duration.				
Badruddin *et al.,* 1997 Pakistan	Diarrhea	‐ ~98% BF continued.	NA	NA	NA	NA	‐ 46% doctors provide no nutritional advice, ‐ Treatment given with expensive medications & intravenous fluids.	NA

Becker *et al.,* 1991 Bangladesh	Diarrhea Fever	NA	NA	‐ Food restriction common	NA	NA	NA	NA
Benakappa & Shivamurthy, 2012 India	ARI Fever	‐ 97% BF continued,	NA	‐ 57% continued CF,	“Special diets”	‐ 19% gave home remedies on elders’ advice.	‐ Doctors asked to avoid "cold" food, like curds, butter milk, fruit juices and bananas in pneumonia (18%). Fever: avoid rice – 15%,	NA
		‐ 3% increased BF,		‐ 12.5% diluted the CF; 70.5% no changes in consistency,	‐ Firmly rooted beliefs about “hot” and “cold“ foods lead to restriction of food available at home,			
		‐ 38.2% decreased BF,						
		‐ 3% stopped BF as they believed that illness would be transmitted to the child,		‐ 43% caregivers reduced CF,				
				‐ Reasons for decreased feeds: child is tired,” or “child cannot digest during illness.	‐ Preferred foods were ‘idli’ (26.41%), rice (18.46%) and bread (16.98%). 17% ‐ no preference. Oily (49%) foods, spicy foods (45.28%) and food considered as "cold" were avoided.			
		‐ Reasons for decreased BF: “child cannot suck”; or ”Mother is sick“.						
							‐ 6% restrict diet to rice and butter milk (diarrhea),	
							‐ 62%: not to restrict any kind of food and to follow the usual diet during recovery.	
Bharti *et al.,* 2006 India	Pneumonia	NA	NA	‐ When children were sick with pneumonia, there were feeding restrictions in a large number of cases.	NA	16.5% sought advice from unqualified practitioners.	NA	NA
Bhuiya & Streatfield, 1995 Bangladesh	Diarrhea Fever	‐ BF is continued in a majority,	NA	‐ ~ 50% continued CF for diarrhea & fever,	NA	‐ Consulting traditional health care providers was quite common.	‐ 100% doctors provide no nutritional advice,	NA
		‐ No mother reported increase in BF,		‐ 39% reduced and 10% discontinued CF,				
		‐ 16% reduced BF,		‐ None gave more CF,		‐ Depending on illness, practitioners advised on foods to eat or restrict.	‐ Advice by doctors mainly on medicine or use of ORS (diarrhea) : 9%,	
		‐ Reduction in BF, highest for fever + cough, fever & diarrhea,		‐ Reasons for discontinuing CF: (1) refusal to eat/ anorexia (2) considered harmful (3) imposition by caregiver,				
							‐ 5‐10% advised to reduce or stop feeding depending on the illness.	
		‐ Reasons to reduce or discontinue BF are: “refusal to eat” or “considered harmful”.		‐ In diarrhea, normal food is believed to be harmful.				
Das *et al.,* 2013 Bangladesh	Diarrhea	NA	‐ 61.3% gave same amount,	‐ No child was given more food,	NA	‐ 39.5% mothers with 0–11 m babies and 20% mothers with 1–2 y children sought advice from uncertified. traditional providers.	NA	NA
			‐ 10.8% offered less,	‐ 71.4% gave same amount,				
			‐ 27.6% gave homemade fluids like thin watery porridge of maize, rice, or wheat, soup, sugar salt water solution & yogurt.	‐ 28.7% gave less food,				
				‐ Younger 0‐1‐y were encouraged to drink/eat more,				
				‐ Few older children (11–24 m) were encouraged to eat more,				
Ansari *et al.,* 2009 Nepal	Diarrhea	NA	Few mothers gave more fluids.	Few mothers gave more food.	NA	NA	NA	NA
Dhadave *et al.,* 2012 India	Diarrhea	‐ 87.2% continued BF,	NA	‐ 78.6% did not restrict CF,	‐ 55.7% of mothers gave home based care.	NA	NA	NA
		‐ 8.5% BF more,		‐ 21.4% restricted CF,				
		‐ 4.3% BF less / stopped.		‐ No mother gave more CF.				
Dongre *et al.,* 2010 India	Diarrhea Fever ARI	‐ 69.7% continued BF.	‐ ~50% continued fluids,	‐ Reduced CF: 50%,	Special diets given to sick children include:	‐ 5.7% mothers went to “faith healers”.	NA	NA
		‐ 73% continued BF children (<1 y) compared to 75% with children >1y,	‐ 43.5% <1y and 53.9% >1 year olds were given extra fluids.	‐ For children with diarrhea only dry food items are given to eat to reduce stress on baby's stomach & the frequency of loose stool,	‐ Food items containing oil & sour food were avoided: leads to difficulty in breathing & cause cough,			
		‐ 73.2% working mothers continued BF.		‐ Few mothers responded that children had reduced appetite & eat less.	‐ A large number of sick children were given herbal tea, honey, ginger etc. to provide relief from cough.			
				‐ More children < 1 y given extra CF compared to children > 2 y,	‐ *Hot* foods (such as papaya, egg, apple, *chikku*) & *cold* foods (such as curd, banana, guava, pomegranate, lemon & custard apple) were avoided,			
				Lower proportion of children from scheduled tribes/ nomadic tribes given increased CF.	Medicines, syrup, injections & tablets are preferred over home remedies.			
Giri & Phalke, 2014 India	Diarrhea	‐ 60% continued BF,	NA	‐ 91% continued CF,	‐ 71%: cold food be restricted in cold/cough,	NA	NA	NA
		‐ 21% decreased BF,		‐ 26.5%: decreased CF,	‐ 89%: curd be restricted in ARI,			
		‐ 17% increased BF,		‐ 9%: stopped CF,	‐ 75%: heavy food be restricted in diarrhea,			
		‐ 2% stopped BF.		‐ Among those who continued: 32% preferred thinner consistency and 8% preferred thick consistency of food.	‐ 72% oily food be restricted in fever,			
					‐ Preferred “special diets” during illness:			
					1) Feeding khichadi (81.5%), milk (67.5%) & biscuits (59%) for ARI,			
					2) Banana (95%), sago (92.5%) & rice water (89%) during diarrhea.			
Gupta & Gupta, 2000 India	Diarrhea Fever	‐ > 75% continued BF,	‐ 92.2%: continued fluids,	NA	‐ Household remedies such as rice gruel, spices, onion juice were adopted by 8.6% mothers.	‐ Mother‐in‐law advised mothers on care of the sick child,	‐ 47.3% mothers prefer private doctors,	NA
		‐ 7.8% stopped BF.	‐ 7.8%: stopped fluids,					
			‐ 8.7% children < 1 years stopped fluids compared to 6.8% children > 1 yr,					
						‐ Home remedies were generally advised.	‐ 20.4% mothers prefer govt doctors,	
							‐ Majority mothers:	
			‐ 15.9% gave home‐based fluids & ORS as first action. These include readymade ORS, sugar‐salt solution, Lassi or Shikkanji.				not given nutritional advice	
							‐ 71% govt. doctors laid emphasis on use of home based fluids/ORS as compared to 27% private practitioners.	
Gupta *et al.,* 2007 India	Diarrhea ARI	NA	‐ 42% : continued fluids,	50%: Complementary feeding (CF) continued	NA	‐ Home remedies were first line of treatment.		NA
			‐ 20% : stopped fluids,	30%: gave home cooked family foods.				
			‐ 42% gave home‐based fluids.					
Hirani, 2012 Pakistan	Diarrhea	NA	NA	‐ CF restriction common	NA	‐ Mothers prefer traditional practitioners.	NA	NA
				‐ For ARI: milk & rice restricted,				
Huffman & Combest, 1990 Bangladesh	Diarrhea	‐ In majority BF continued	NA	‐ >75%infants often refuse other foods.	NA	NA	NA	NA
Kasi et al, 1995 Pakistan	Diarrhea	NA	NA	NA	NA	NA	‐ 71% doctors gave no nutritional advice instead prescribed drugs & ORS, ‐ 46% doctors provided advice on BF and not CF, ‐ 7% introduction of CF after illness.	NA
Kaur et al, 1994 India	Diarrhea	‐ 85.5% continued BF, ‐ Very few stopped BF.	‐ 95.8% : Fluids continued, ‐ 39.6 %: Fluids usual amounts, ‐ 50%: Fluids restricted, ‐ 4.1%: Fluids stopped.	‐ ~60%: usual amounts CF, ‐ 35.4%: restricted CF, ‐ ~33% stopped CF due to less appetite, ‐ Diets of sick children were modified.	NA	NA	NA	‐ CF restriction favored by 98.1% earlier, now is favored by 35%.Very few withheld BF.
Kaur & Singh, 1994 India	Diarrhea	NA	‐ Homemade fluids given, ‐ Few restricted fluids.	‐ 38.2% : same amount CF, ‐ 61.8%: CF restricted.	NA	NA	NA	‐ Health education programme: Giving salt sugar solution increased from 2% to 29.6%; only 23.8% gave 3‐4 times/day, ‐ Less CF improved from 55% to 29%.
Kaushal et al, 2005 India	Diarrhea Fever	‐ >75% continued BF, ‐ Refusal to feed was considered “normal during illness” & as a marker of a sickness by most grandmothers & mothers. They believed that health‐seeking for poor feeding could be delayed for 1 day.	NA	NA	NA	‐ Grandmothers influenced mother's caregiving practices, ‐ They advised mothers on home‐based care and patterns of feeding, ‐ Helped mothers to recognize danger signs such as poor activity, poor feeding, hypothermia, and respiratory distress, ‐ Grandmothers had homemade remedies for common ailments, some of which could be harmful.	NA	NA
Malik et al, 1991 Pakistan	Diarrhea	‐ 70% continued BF,	NA	‐ 78%‐87%: were given normal family foods.	Sick children also received solid & semi‐solid diet which was either "Khitchri" or banana as mentioned by more than half of the respondents.	NA	NA	NA
Mangala et al, 2000 India	Diarrhea	‐ 92.4% continued BF, ‐ 15.1% BF more frequently, ‐ 8.6% ceased BF.	‐ 47% aware of increased fluids.	‐ 30%: Continued CF, ‐ 2.4%: gave “special diets” i.e., cooking practices modified i.e., food mashed or ground food for easier digestion (modification in food quality), ‐ 16.7% more CF, ‐ Educational intervention: increase of mothers modifying food to make it soft & more easily digestible 2.4% to 26.2% ‐ Increased feeding after illness: None.	NA	NA	NA	After an educational intervention improvements in ‐ BF frequency (15.1% to 47.2%) ‐ Modification of CF preparation (2.4% to 26.2%) ‐ Increased CF after illness (1.2% to 20%)
Memon et al, 2010 Pakistan	Diarrhea ARI Fever	NA	‐ Most mothers continued fluids, ‐ 40%: aware of increased fluids.	‐ 53%: gave more CF as home cooked meals.	NA	NA	NA	NA
Mishra et al, 1990 India	Diarrhea	NA	NA	‐ 60‐66% CF was given as usual, ‐ 27‐30% cases CF modified to make food soft as normal foods can trigger diarrhea, ‐ 6.25% CF was stopped to help child recuperate and restricting food reduces stools.	‐ 27% to 30% cases: special diets were given to sick children, ‐ “Hot” foods are avoided.	NA	NA	NA
Piechulek et al, 1999 Bangladesh	Diarrhea & ARI	‐ BF continued in a majority, ‐ 22.2% discontinued BF for diarrhea, ‐ Reasons for not BF & giving animal milk: 1) Mother's belief that fluids are harmful, cannot be absorbed. Another reason for restricting breastmilk 2) Improvements seen in diminished stool volume.	Diarrhea ‐ None: Increased fluids, ‐ 91.5%: continued fluids, ‐ 0.8%: stopped fluids, ‐ 87% Fluid restriction (diarrhea) and 9.3% (pneumonia). ARI ‐ 14.8%: Restricted fluids until full recovery.	‐ 38%: continued CF, ‐ None: increased CF, ‐ >54.6%: restricted/withheld CF, ‐ 59.1% restricted for diarrhea & 22.4% for pneumonia, ‐ ~30%: stopped CF for > 24 hrs; of this a small proportion stopped until child's recovery, ‐ Mothers withhold food because 1) medical advice; 2) own belief of “keeping bowels at rest”; 3) poor appetite, ‐ Educated mothers less likely to withhold food, ‐ Food quality modified to “cure illness”.	‐ 97% mothers gave special diet to their ill child. According to type of illness, certain foods were avoided or preferred, ‐ In all illnesses foods like fish, meat & vegetables are “avoided” as they increase loose motions or prolong disease effects, ‐ Special diet to cure cold: warm milk & a syrup of basil “tulsi” leaves (hot foods), ‐ Special foods to cure ARI/pneumonia: foods that aggravate symptoms of cold like fish, duck or pigeon meat; and banana, green papaya, green coconut and some vegetables are avoided, ‐ Special foods to cure diarrhea: raw banana & coconut reduce abdominal discomfort & diminish frequency of stools, ‐ Fish, milk, meat, or vegetables avoided by ~98% others as they “increase the frequency of loose motions” (diarrhea).	‐ NA	‐ Many mothers withheld food because of doctors’ advice.	NA
Rashid et al, 2001 Bangladesh	ARI	NA	NA	‐ CF restricted and given once a day.	‐ Most mothers modified children's diets, ‐ ‘Cold foods’ i.e., left over or stale foods were avoided as “aggravate pneumonia”, ‐ Once a day: only rice & salt or dry bread was provided and are deprived of vegetables & fruits.	‐ 16% mothers were advised by mothers‐in law to restrict certain types of food, ‐ Traditional and allopathic care was sought depending on the perceived severity of the illness.	NA	NA
Shah et al, 2011 India	Diarrhea	‐ 50% BF continued, ‐ 49.3% decreased/stopped, ‐ Reasons for stopping BF: Energy dense foods that the mothers consume is secreted in the breastmilk that the child cannot digest.	NA	NA	NA	29% mothers consulted traditional medical practitioners or quacks.		
Sharma & Thakur, 1995 India	Diarrhea Fever	NA	NA	‐ CF is commonly restricted.	‐ CF quality is modified, ‐ Foods preferred during cough and fever: Cold & light foods i.e., curd, fruits, rice, sago, barley, and biscuits.		NA	NA
Singh et al, 1994 India	Diarrhea	‐ BF continued in most cases.	NA	NA	‐ Feeding was not withheld but changes made in the nature of foods given which varied by illness type, Special diets given in diarrhea: ‐ 1) daliya & khitchri because ‘intestines become weak & children are unable to digest heavy foods”; ‐ 2) Diluted tea & banana to frequency of stools; ‐ 3) Cow's milk as “evil eye had contaminated breast milk. ‐ 4) Foods avoided are “wheat flour bread” & milk as it is “too heavy”; and “Hot foods' like apple, mango, jaggery, nearly all pulses as these could enhance the frequency & intensity of diarrhea.	NA	NA	NA
Zeitlyn et al, 1993 Bangladesh	Diarrhea	NA	NA	‐ >36%: restrict CF and 64% gave normal home cooked meals, ‐ Withholding food is a first measure to treat diarrhea, ‐ 10% stopped CF to give bowels a rest, ‐ <25%: gave CF as usual. ‐ Food is restricted as mothers recognize that children have reduced appetites & are reluctant to force feed to eat. Working mothers do not want to force feed the child due to “time constraints” ‐ Soft foods given children <10‐months as mothers believe “illness weakens a child's digestive power & soft diets in the form of gruels & soups are easier to digest”.	‐ Special diets: Normal family diets are modified to soft foods to aid digestion, ‐ Foods are restricted/modified due to cultural notions “digestive power in illness”, ‐ Soft foods given children <10‐months as mothers believe “illness weakens a child's digestive power & soft diets in the form of gruels & soups are easier to digest”, ‐ Maternal or family's perceptions of “hot or cold foods” and its perceived beneficial/ harmful effects. ‐ Fish is avoided: vehicle attracting “evil” forces that perpetuate illness. ‐ Cold & stale foods i.e., foods cooked several hours earlier are considered breeding grounds for bacteria.	‐ A few mothers reported traditional practitioners advised them to withhold foods when their child had diarrhea, including breastmilk, ‐ Mothers in law was the main source of advice to mothers on home remedies.	‐ Health providers provided nutrition related advice on feeding a sick child during illness, ‐ A few mothers reported that health providers advised them to withhold foods when their child had diarrhea, including breastmilk.	

BF, breast feeding; ARI, acute respiratory inspection; NA, not applicable.

### Research evidence on caregivers' behaviours and health providers' practices on infant and young child feeding during and after common childhood illnesses

The bibliographic search identified 32 peer‐reviewed publications that met the inclusion criteria for this review. One study (3%) was from Nepal, eight studies (25%) were from Bangladesh, seven studies (22%) were from Pakistan and 15 studies (47%) were from India.

The majority of the studies (*n* = 31; 97%) reported IYCF practices during common childhood illness, while only one study reported IYCF practices both during and after illness.

Thirty studies (94%) reported IYCF practices for children with diarrhoea, eight studies (25%) reported IYCF practices for children with pneumonia and five studies (16%) reported IYCF practices for children with fever. Most studies (*n* = 29; 91%) reported caregivers' IYCF behaviours when children were sick, while only six studies (19%) reported health providers' IYCF counselling to mothers of sick children. Twenty‐eight (88%) were observational studies. Only four (13%) studies – one in Bangladesh and three in India – assessed the impact of one or more interventions to improve IYCF practices during/after illness.

The findings of our review are organized around the seven key focus areas (Table 8).

#### Breastfeeding during and after common childhood illnesses

Sixteen studies (50%) investigated whether children continued to be breastfed while they were sick. All the studies reported that most mothers (range 69.7–98.0%) continued to breastfeed their sick children irrespective of children's age or the nature of their illness (Huffman & Combest 1990; Malik *et al*. 1991; Badruddin *et al*. 1991, 1997; Kaur *et al*. 1994; Singh 1994; Bhuiya & Streatfield 1995; Piechulek *et al*. 1999; Gupta & Gupta 2000; Mangala *et al*. 2000; Kaushal *et al*. 2005; Shah *et al*. 2011; Benakappa & Shivamurthy 2012; Dhadave *et al*. 2012; Dongre *et al*. 2010; Giri & Phalke 2014). Three studies reported that some mothers (range 8.5–17.0%) breastfed their children more frequently when children were sick (Mangala *et al*. 2000; Dhadave *et al*. 2012; Giri & Phalke 2014); conversely, four studies reported that some mothers (range 4.3–49.3%) breastfed their sick children less frequently (Piechulek *et al*. 1999; Shah *et al*. 2011; Benakappa & Shivamurthy 2012; Giri & Phalke 2014); lastly, six studies reported that some mothers (range 1–9%) ceased to breastfeed when children were sick (Kaur *et al*. 1994; Gupta & Gupta 2000; Mangala *et al*. 2000; Shah *et al*. 2011; Dhadave *et al*. 2012; Giri & Phalke 2014). The three main reasons given by mothers for reducing or ceasing breastfeeding while children were sick are: (1) the belief that infants could not digest breast milk when they were sick (two studies; Piechulek *et al*. 1999; Shah *et al*. 2011); (2) the perception that children were anorexic/had no appetite and/or refused to be fed (two studies; Bhuiya & Streatfield 1995; Benakappa & Shivamurthy 2012); and/or (3) the belief that breast milk had become harmful to the child because of mystical/evil forces and/or that the illness had been transmitted by the mother to the child through mother's milk (three studies) (Bhuiya & Streatfield 1995; Kaushal *et al*. 2005; Benakappa & Shivamurthy 2012). Two studies reported that a significant proportion of mothers (range 35–61%) – particularly among those with young infants 0–11 months old and/or children with diarrhoea – switched back to predominant or exclusive breastfeeding when children were sick (Ahmed *et al*. 1992; Shah *et al*. 2011).

#### Fluid intake during and after common childhood illnesses

Ten studies (31%) investigated whether children continued to be given fluids when they experienced common illnesses and/or whether fluid intake increased or decreased when children were sick. Nine studies reported that most mothers (range 40–92%) continued to administer fluids to their sick children (Kaur & Singh 1994; Kaur *et al*. 1994; Piechulek *et al*. 1999; Gupta & Gupta 2000; Agha *et al*. 2007; Gupta *et al*. 2007; Dongre *et al*. 2010; Memon *et al*. 2010; Das *et al*. 2013). Two studies reported that some mothers (range 6–28%) gave additional liquids/fluids to their children during illness (Kaur *et al*. 1994; Das *et al*. 2013). Three studies reported that, in addition to water, mothers fed sick children home‐made fluids such as watery porridges made from maize, rice or wheat; soups; sugar–salt–water solutions; and/or yogurt (Gupta & Gupta 2000; Gupta *et al*. 2007; Das *et al*. 2013). Six studies reported that mothers restricted the amount of liquids/fluids given to sick children (Kaur & Singh 1994; Kaur *et al*. 1994; Piechulek *et al*. 1999; Agha *et al*. 2007; Ansari *et al*. 2009; Das *et al*. 2013). Fluid restriction was more frequent during diarrhoea episodes (range 4–87%) (Kaur & Singh 1994; Kaur *et al*. 1994; Piechulek *et al*. 1999; Agha *et al*. 2007; Das *et al*. 2013) than during episodes of fever or pneumonia (range 8.3–15%) (Piechulek *et al*. 1999; Gupta & Gupta 2000; Agha *et al*. 2007). Only one study reported the reasons given by mothers for restricting children's fluid intake during sickness (Piechulek *et al*. 1999); these were: (1) the belief that fluids could not be absorbed during diarrhoea and thus were harmful; and (2) the perception that a reduction in the stool volume in children with diarrhoea was an improvement of the child's condition. Two studies reported that the proportion of mothers who were aware that children need more fluids during sickness ranged between 40% and 47% (Mangala *et al*. 2000; Memon *et al*. 2010).

#### Complementary foods and feeding practices during and after common childhood illnesses

Twenty studies (63%) investigated whether children were fed lower, similar or larger amounts of soft, semi‐solid or solid foods when they suffered from common childhood illnesses. Thirteen studies reported that children (range 25–79%) continued to be fed regular family foods as usual, with no restrictions/changes in frequency and/or quantity (Becker *et al*. 1991; Badruddin *et al*. 1991; Malik *et al*. 1991; Ahmed *et al*. 1992; Zeitlyn *et al*. 1993; Kaur *et al*. 1994; Bhuiya & Streatfield 1995; Gupta *et al*. 2007; Mangala *et al*. 2000; Dongre *et al*. 2010; Memon *et al*. 2010; Dhadave *et al*. 2012; Das *et al*. 2013).

However, a significant number of studies (18) indicated that feeding restrictions during common childhood illnesses were frequent; these restrictions affected feeding frequency (4 studies: Mangala *et al*. 2000; Dongre *et al*. 2010; Benakappa & Shivamurthy 2012; Giri & Phalke 2014); food quality (11 studies: Mishra *et al*. 1990; Badruddin *et al*. 1991; Ahmed *et al*. 1992; Zeitlyn *et al*. 1993; Sharma & Thakur 1995; Piechulek *et al*. 1999; Mangala *et al*. 2000; Agha *et al*. 2007; Dongre *et al*. 2010; Benakappa & Shivamurthy 2012; Giri & Phalke 2014); and/or food quantity (7 studies: Kaur *et al*. 1994; Bhuiya & Streatfield 1995; Piechulek *et al*. 1999; Mangala *et al*. 2000; Agha *et al*. 2007; Dhadave *et al*. 2012; Das *et al*. 2013). Food restrictions seemed to be more common during diarrhoea episodes (up to 83% of the mothers interviewed) than during episodes of pneumonia and fever (up to 70% and 47% of the mothers interviewed, respectively). The reasons most commonly reported by mothers for restricting food intake when children were sick were: (1) caregivers' perception that children had less appetite or refused to eat/be fed (five studies: Zeitlyn *et al*. 1993; Kaur *et al*. 1994; Bhuiya & Streatfield 1995; Piechulek *et al*. 1999; Dongre *et al*. 2010); (2) mothers' reluctance to ‘force’ the child to eat (two studies: Zeitlyn *et al*. 1993; Bhuiya & Streatfield 1995); (3) mothers' inability to feed the children more food/more frequently owing to resources (fuel) or time constraints (one study: Zeitlyn *et al*. 1993); (4) mothers' belief that illness ‘disturbed’ the digestive system and that feeding ‘normal’ foods was harmful to the sick child as the child's digestive power was ‘diminished’ and ‘normal foods’ would trigger diarrhoea, produce cough and/or put stress on the child's stomach (nine studies: Mishra *et al*. 1990; Ahmed *et al*. 1992; Zeitlyn *et al*. 1993; Bhuiya & Streatfield 1995; Sharma & Thakur 1995; Mangala *et al*. 2000; Dongre *et al*. 2010; Benakappa & Shivamurthy 2012; Giri & Phalke 2014); (5) mothers' belief that withholding certain foods would help to cure diarrhoea whereas introducing normal foods before the child was cured would have a detrimental effect on the development of the child or would lead to a ‘big belly’ (three studies: Mishra *et al*. 1990; Ahmed *et al*. 1992; Zeitlyn *et al*. 1993); and/or (6) the belief that restricting food intake was a first measure to manage diarrhoea at home and reduce the frequency of loose stools (five studies: Mishra *et al*. 1990; Zeitlyn *et al*. 1993; Piechulek *et al*. 1999; Dongre *et al*. 2010). Two studies that measured food intake in sick children 6–23 months old reported that the mean energy intake of children was significantly lower than that of healthy children and up to 70% below WHO recommendations (Becker *et al*. 1991; Benakappa & Shivamurthy 2012). Two studies reported that some mothers (range 10–30%) ceased feeding their child for 24 h or longer following medical advice, because mothers perceived that the children had poor/no appetite and/or because social norms advised ‘to keep bowels at rest’ (Zeitlyn *et al*. 1993; Piechulek *et al*. 1999). None of the studies reported an increase in children's food intake (frequency, quantity and/or quality). Only four studies assessed mothers' knowledge about the feeding needs of sick children; few mothers (range 17–38%) recognized the importance of feeding sick children nutritious diets comprising vegetables, pulses, small fish and/or other nutrient‐rich foods (Zeitlyn *et al*. 1993; Piechulek *et al*. 1999; Agha *et al*. 2007; Benakappa & Shivamurthy 2012).

#### Traditional beliefs and their role in IYCF during and after common childhood illnesses

Thirteen studies (41%) explored the importance of traditional beliefs and perceptions on IYCF practices during and after common childhood illnesses. Nine studies reported that when children were sick, caregivers (range 13–98%) replaced children's usual diets with ‘special diets’ owing to the belief that children's usual diets need to be modified to aid digestion ‘because intestines become weak’ (Mishra *et al*. 1990; Ahmed *et al*. 1992; Zeitlyn *et al*. 1993; Dongre *et al*. 2010; Benakappa & Shivamurthy 2012; Giri & Phalke 2014). Young children were often fed home remedies, herbal medicines and teas, and ‘soft foods’ in the form of soups and gruels (Badruddin *et al*. 1991; Zeitlyn *et al*. 1993; Piechulek *et al*. 1999; Gupta & Gupta 2000; Dongre *et al*. 2010; Benakappa & Shivamurthy 2012; Giri & Phalke 2014) because they were perceived to ‘be lighter on the stomach’, ‘be easier to digest’, ‘reduce abdominal pain’ and/or ‘diminish the frequency of stools’. Conversely, foods like fish, milk, meat, food items containing oil or even vegetables were avoided because they were considered ‘difficult to digest’ or ‘too heavy to digest’ (Ahmed *et al*. 1992; Zeitlyn *et al*. 1993; Piechulek *et al*. 1999; Giri & Phalke 2014).

Seven studies (Mishra *et al*. 1990; Zeitlyn *et al*. 1993; Piechulek *et al*. 1999; Rashid *et al*. 2001; Dongre *et al*. 2010; Benakappa & Shivamurthy 2012; Giri & Phalke 2014) reported that caregivers avoided giving specific ‘hot’ or ‘cold’ foods to sick children because these foods were considered inappropriate for certain diseases. Foods commonly avoided in case of diarrhoea were eggs/meat (range 25% to >95%), roti/chapatti/wheat flour breads (~70%) and milk (47–50%); in case of pneumonia, commonly avoided foods were fish/duck/pigeon (>90%) or curd/buttermilk (range 76% to 93%); in case of fever, commonly avoided foods included rice (40%) and curd/butter milk (range 60–70%). Two studies reported that some caregivers (range 16–23%) avoided giving sick children foods like fish, meat or eggs because ‘they attract evil forces’ and thus they were harmful to children (Zeitlyn *et al*. 1993; Piechulek *et al*. 1999).

#### Community elders/traditional practitioners' advice on IYCF during/after childhood illnesses

Five studies (16%) reported on the role of mothers‐in‐law (four studies: Zeitlyn *et al*. 1993; Rashid *et al*. 2001; Kaushal *et al*. 2005; Gupta *et al*. 2007) or other family elders (Ahmed *et al*. 1992) on decision regarding IYCF practices when children were sick. Common advice given by family elders to mothers when children were sick included the following: (1) to opt for home remedies as a first line of treatment (Zeitlyn *et al*. 1993; Gupta *et al*. 2007); (2) to accept children's refusal to eat/be fed as ‘normal’ and delay feeding by one day (Kaushal *et al*. 2005); and/or (3) to refrain from giving sick children certain foods such as fish, meat, vegetables or milk (Zeitlyn *et al*. 1993). Nine studies (28%) reported that a varying proportion of mothers (range 5.3–84%) sought help from traditional/unqualified practitioners when their children were sick (Zeitlyn *et al*. 1993; Kaur *et al*. 1994; Bhuiya & Streatfield 1995; Rashid *et al*. 2001; Bharti *et al*. 2006; Dongre *et al*. 2010; Shah *et al*. 2011; Hirani 2012; Das *et al*. 2013). However, only one study described the role of traditional/unqualified practitioners on IYCF counselling to mothers when children were sick (Bhuiya & Streatfield 1995). This study reported that traditional/unqualified practitioners advised mothers to restrict children's food intake; however, it did not provide specific details on the types of foods that a mother should avoid when her child was sick.

#### Health professionals' advice on IYCF during and after common childhood illnesses

Six studies (19%) investigated the role of health professionals in providing IYCF advice to mothers when children were sick (Zeitlyn *et al*. 1993; Bhuiya & Streatfield 1995; Kasi *et al*. 1995; Badruddin *et al*. 1997; Piechulek *et al*. 1999; Benakappa & Shivamurthy 2012). In general, health professionals gave little or no advice to mothers on how to feed their children during or after the illness episode: three studies reported that health providers (range 46–100%) advised mothers to continue breastfeeding (Bhuiya & Streatfield 1995; Badruddin *et al*. 1997; Piechulek *et al*. 1999); two studies reported that health providers (range 9–71%) advised mothers to give oral rehydration solution/home‐based fluids to infants and young children suffering from diarrhoea (Bhuiya & Streatfield 1995; Kasi *et al*. 1995). No study reported that health providers advised mothers to increase fluid intake when children were sick. Similarly, no study reported that health providers advised mothers to encourage their sick children to eat soft, varied and favourite foods during illness, as recommended by WHO (WHO 2003a, 2003b). Conversely, two studies (Zeitlyn *et al*. 1993; Bhuiya & Streatfield 1995) reported that health providers advised mothers (proportion not reported) to withhold breast milk and foods such as rice and buttermilk (in case of diarrhoea), rice (in case of fever) or *cold foods* like curd, buttermilk, fruit juices and bananas (in case of pneumonia or acute respiratory infections). Two studies reported that health professionals (range 7–62%) advised mothers to feed their children soft and semi‐solid foods only after children had recovered from the illness (Kasi *et al*. 1995; Benakappa & Shivamurthy 2012).

#### Interpersonal and group counselling on IYCF during/after common childhood illnesses

Three studies (9%) – all in India – reported the impact of behaviour change communication interventions on mothers' IYCF practices when children had diarrhoea (Kaur & Singh 1994; Kaur *et al*. 1994; Mangala *et al*. 2000). Two studies reported the impact of home visits and group counselling by trained community health workers. In these studies, the proportion of mothers giving ‘less than usual’ amounts of food to their sick children declined from 98% to 35% and from 55% to 29% (Kaur & Singh 1994; Kaur *et al*. 1994). The third study assessed the impact of cooking demonstrations using locally available foods and interpersonal counselling sessions on mothers' IYCF practices when children were sick (Mangala *et al*. 2000). The results of the intervention indicated the following: (1) the proportion of mothers who breastfed more frequently while children had diarrhoea increased from 15% to 47%; (2) the proportion of mothers who modified family foods to make them soft and digestible (i.e. more palatable) increased from 2% to 26%; and (3) the proportion of mothers who fed their children additional food for at least 2 weeks after the diarrhoea episode increased from 0% to 20%.

### Review of national policy and programme frameworks for infant and young child feeding during and after common childhood illnesses

We reviewed national policy and programme documents to assess whether national frameworks for maternal and child nutrition integrate IYCF during and after illness. In addition, we conducted interviews with 13 key informants to document the existing national programmes that protect, promote and support optimal IYCF practices for children during and after illness (Table 9).

**Table 9:** Policies and programmes related to infant and young child feeding (IYCF) during and after illness in South Asian countries (1990–2014)

	Afghanistan	Bangladesh	Bhutan	India	Nepal	Maldives	Pakistan	Sri Lanka
A national stand‐alone policy for the protection, promotion and support of optimal IYCF practices is available	Y	X	Y	Y	X	X	X	Y
A national nutrition and/or food security policy that includes IYCF is available	Y	X	Y	X	Y	X	X	X
The national IYCF/national nutrition/food security policy includes IYCF during and after illness	X	X	X	Y	Y	X	X	X
A national programme for the protection, promotion and support of optimal IYCF practices exists	Y	Y	Y	Y	Y	X	Y	Y
National guidelines for the protection, promotion and support of optimal IYCF practices are available	Y	Y	Y	Y	Y	Y	Y	Y
National guidelines for the protection, promotion and support of optimal IYCF include IYCF during and after illness	X	X	Y	Y	X	Y	Y	Y
The national training package for IYCF protection, promotion and support includes IYCF during/after illness	X	Y	Y	Y	Y	X	X	Y
A national programme for the integrated management of childhood illness (IMCI) exists	Y	Y	Y	Y	Y	Y	Y	Y
The national guidelines for IMCI include guidance on feeding children when they are sick	Y	Y	Y	Y	X	Y	Y	Y
The national guidelines for IMCI include guidance on feeding children after being sick	Y	Y	Y	Y	X	Y	Y	Y
The national training package on IMCI includes guidance on IYCF during and after illness	X	Y	X	Y	X	Y	X	Y

Y: Yes, X: No

Five of the eight countries have a national IYCF policy, either as a stand‐alone policy framework on infant feeding or as part of a larger policy framework on nutrition/food security. However, only two countries – India and Nepal – have integrated the feeding needs of children during and after illness in their IYCF policy framework.

All countries have national guidelines on IYCF, and seven countries have a national programme for the protection, promotion and support of optimal IYCF. However, only five countries include in their national IYCF guidelines guidance on how children should be fed during and after illness, and only five countries have developed a training package on IYCF for programme staff that includes IYCF for children during and after illness.

All countries have a national programme for the integrated management of childhood illnesses (IMCI); six countries have national IMCI guidelines that include guidance on feeding children during and after illness; however, only four countries have developed an IMCI training package that includes guidance on how to feed children when they are sick and after being sick.

## Discussion

We conducted a comprehensive review of the available evidence on IYCF practices during and after common childhood illnesses – diarrhoea, fever and pneumonia – in South Asia (1990–2014) to inform policy formulation, programme design, advocacy and research prioritization to protect, promote and support optimal IYCF practices during and after common childhood illnesses in South Asia post 2015.

Demographic Health Survey data on IYCF during common childhood illnesses were available only for Bangladesh, India, Nepal and Pakistan, which are home to ~96% of the children under 5 years of age in South Asia (UNICEF 2015). Similarly, the 32 publications that met the inclusion criteria of our review focused on these four countries. Furthermore, the available DHS data in these four countries were limited to IYCF during diarrhoea episodes. No survey data were available on IYCF practices after episodes of diarrhoea or during/after episodes of fever or pneumonia. Similarly, the published research was primarily focused on IYCF practices during diarrhoea and to a lesser extent during fever or pneumonia episodes. Research evidence on IYCF after common childhood illnesses was practically inexistent.

Demographic Health Survey data indicate that in the countries included in the analysis, children 0–23 months old suffer from common childhood illnesses frequently, as nearly one‐third of the mothers/caregivers reported that their children had suffered from diarrhoea or pneumonia in the 2 weeks prior to the survey. These findings are in line with reports indicating that 39% of child deaths in South Asia are due to diarrhoea and/or pneumonia (UNICEF 2012). Importantly, DHS data indicate that in all the countries included in our review, the occurrence of diarrhoea, pneumonia and fever was lowest during the exclusive breastfeeding period (0–5 months) and highest during the early complementary feeding period (6–11 months). This is most likely due to the well‐documented protective benefits of exclusive/predominant breastfeeding in the first 6 months of life and the higher levels of infection in late infancy and early childhood due to children's increased intake of complementary foods and fluids that may be contaminated as well as the ingestion of faecal bacteria through mouthing soiled fingers or household items when children begin to crawl and explore their environment (Kosek *et al*. 2003; Dewey & Mayers 2011; WHO 2013).

Our review shows that in South Asia, IYCF behaviours and practices during common childhood illnesses are far from optimal. Most infants and young children continue to be breastfed when they are sick; however, few children (<20%) are breastfed more frequently as recommended. Studies in other settings have reported a similar practice, as most mothers continue to breastfeed their sick children without altering the number of nursing episodes, total amount of time of suckling or energy derived from breast milk (Hoyle *et al*. 1980; Brown *et al*. 1990; Martz & Tomkins 1995; Brown 2003).

Similarly, most sick children continue to be fed fluids. However, few children (range 7.4–21.7% in Pakistan and Bangladesh, respectively) were fed fluids more frequently as recommended. Mothers' awareness about children's need for more fluids during sickness is low. This evidence is in line with reports indicating that in developing countries, less than a quarter (22%) of children are fed more fluids during illness (UNICEF/WHO 2009). Conversely, a significant proportion of mothers/caregivers in Bangladesh (26.7%), Pakistan (37.9%) and India (42.2%) fed their sick children less fluids than usual or no fluids at all in contrast with reports from other countries (Bani *et al*. 2002; Saha *et al*. 2013).

We find that food restrictions are frequent. Many children were fed lower quantities and/or less frequently when they were sick. As many as 36% of mothers/caregivers in Bangladesh, 41% in Pakistan and 43% in India reported that they fed their children less food than usual or no food at all during the last diarrhoea episode. Only one‐third (34%) of the mothers/caregivers in India to about half (55%) in Nepal reported that they fed their children same/similar amounts of food as usual during the diarrhoea episode. Studies in Latin America have indicated that anorexia is an important factor in the reduction of children's dietary intake during illness (particularly when diarrhoea or fever are present) as mothers/caregivers tend to give in when sick children send a ‘food reject’ signal (Bentley *et al*. 1991, 1995). The combined effects of anorexia and tradition‐driven withdrawal of complementary feeding during common childhood illnesses can be devastating (Scrimshaw & Sangiovanni 1997).

Our review indicates that in South Asian countries, mothers'/caregivers' knowledge about the feeding needs of sick children is limited and that feeding practices are often guided by traditional beliefs and norms that encourage the use of ‘special’ foods/diets to replace ‘usual diets’ when children are sick. Similarly, many caregivers seem to avoid giving certain ‘hot’ or ‘cold’ foods to sick children because these foods are considered inappropriate for specific diseases. Studies have reported that deeply held beliefs and traditions determine the types of foods or preparation methods that are ‘healthy’ or ‘unhealthy’ for sick children, when and what types of complementary foods are given to children and how to feed children who are sick and/or do not want to eat. These beliefs are heavily influenced by the individuals who surround mothers – that is, husbands, mothers‐in‐law, grandmothers and other family/community members – and the health care providers upon whom caregivers depend for support (Martz & Tomkins 1995; Stewart *et al*. 2013).

Care‐seeking practices in South Asia are said to be below global estimates for low‐income and middle‐income countries (Walker *et al*. 2012). However, the latest DHS data available for the countries included in our review indicate that a significant proportion of mothers/caregivers (ranging from 30% to 84% depending on country and illness) took their sick children for medical advice during the last episode of diarrhoea, fever or pneumonia. Our review shows that few published studies have investigated the quality of health providers' counselling on IYCF to mothers/caregivers when children are sick. The evidence reviewed indicates that when mothers/caregivers seek advice/support in the primary health care system, health professionals provide little or no advice to mothers/caregivers on how to feed children when they are sick/convalescent. In general, health providers do not advise mothers to increase children's fluid intake and encourage sick children to eat soft, varied and favourite foods during illness, while increasing breastfeeding frequency as is recommended. Moreover, there is indication that a non‐negligible proportion of health providers advise mothers to withdraw breast milk and/or specific nutritious foods/all complementary foods until children recover from illness.

Studies in other low‐income and middle‐income countries have found the following: (1) health workers do not maximize their contacts with women and children to support optimal IYCF; (2) there is poor knowledge among health practitioners on how to feed and/or manage sick children and manage children with poor appetite; (3) even when a national normative and guidance frameworks on IYCF for sick children are in place, a limited proportion of paediatricians and family practitioners follow them; and (4) the quality of care and advice among private practitioners is not necessarily better than among public health system providers (Bezerra *et al*. 1992; Bojalil *et al*. 1998; Baker *et al*. 2013; Lutter *et al*. 2013).

## Conclusion

Diarrhoea and pneumonia remain the leading infectious causes of childhood morbidity and mortality in South Asia (Fischer *et al*. 2013). Compelling evidence indicates that childhood diarrhoea and pneumonia deaths are avoidable and that scaling up optimal feeding behaviours and practices in combination with appropriate case management can avoid most of these deaths (Bhutta *et al*. 2013).

Our review shows that information of IYCF behaviours and practices during illnesses in South Asia is limited while information of IYCF after common childhood illnesses is virtually inexistent. The evidence reviewed indicates that in South Asia, IYCF behaviours and practices during common childhood illnesses are far from optimal. In general, sick children continue to be breastfed. However, few are breastfed more frequently to compensate for the additional fluid and nutrient requirements associated with illnesses, while a significant proportion of children is breastfed less frequently than usual. Restriction or withdrawal of complementary foods during illness is frequent because of children's anorexia (perceived or real), poor awareness by caregivers' about the feeding needs of sick children, traditional beliefs and behaviours, and/or suboptimal counselling and support by health workers. As a result, many sick children are fed less frequently and/or lower quantities of complementary foods.

Mothers/caregivers often turn to family/community elders and traditional/non‐qualified practitioners to seek advice on how to feed their sick children. Thus, traditional beliefs and behaviours often guide the use of ‘special’ feeding practices, foods and diets for sick children. Our review indicates that when children are sick, a significant proportion of families turn to the primary health care system for advice and support. In general, health professionals give little or no advice to mothers/caregivers on how to feed their children while they are sick. However, the few intervention studies available indicate that inter‐personal and group counselling as part of primary health care can substantially improve mothers' IYCF knowledge and practices during common childhood illnesses.

Global guidance and normative frameworks are in place to address the feeding of sick children during and after illness (WHO 2003a, 2003b; WHO/UNICEF 2003; WHO 2005). All the countries included in our review have national guidelines on IYCF and a national programme for the IMCI. However, there seem to be important policy, guidance and capacity building gaps in these frameworks with respect to IYCF when children are sick or convalescent. Our review indicates that a limited proportion of health practitioners follows all aspects of these guidance.

In light of our findings, it seems reasonable to recommend the following as a way forward to protect, promote and support optimal IYCF practices during and after common childhood illnesses in South Asia post 2015:

1. align national policy frameworks and programmatic guidance with internationally agreed upon recommendations on IYCF during and after common childhood illnesses, with a particular emphasis on diarrhoea, fever and pneumonia;
2. expand the DHS and National Nutrition Surveys to include quantitative information on IYCF during and after common childhood illnesses, with appropriate geographic, socio‐economic and gender disaggregation;
3. collect qualitative and quantitative information on caregivers' behaviours and health workers' practices related to IYCF during and after common childhood illnesses to identify the most important drivers of current behaviours/practices and bottlenecks to optimal IYCF when children are sick/convalescent;
4. build the capacity of facility‐based and community‐based health workers to provide mothers/caregivers with timely and accurate information, counselling and support on IYCF when children are sick/convalescent;
5. design and implement effective communication strategies that combine interpersonal communication and mass communication to address harmful beliefs and norms with respect to the nutrient and feeding needs of children during/after common illnesses; and
6. document the effectiveness, impact and lessons learned of the capacity building and communication strategies to improve IYCF during and after common childhood illnesses and their implications for programme scale up and universalization.

## Source of funding

The UNICEF Regional Office for South Asia provided support for data analysis and paper writing. This research received no specific grant from any funding agency in the commercial sector.

## Conflicts of interest

The authors declare that they have no conflicts of interest. The opinions expressed on this paper are those of the authors and do not necessarily represent an official position of UNICEF.

## Contributions

VMA designed the study, KP led data analysis. Both authors contributed equally to data interpretation and manuscript writing and have read and approved the final submission.
